# Bacteremia causes hippocampal apoptosis in experimental pneumococcal meningitis

**DOI:** 10.1186/1471-2334-10-1

**Published:** 2010-01-03

**Authors:** Christian Østergaard, Stephen L Leib, Ian Rowland, Christian T Brandt

**Affiliations:** 1 Department of Clinical Microbiology, Copenhagen University Hospital Herlev, Herlev, Denmark; 2 National Center for Antimicrobials and Infection Control, Statens Serum Institute, Copenhagen, Denmark; 3 Institute for Infectious Diseases, University of Bern, Bern, Switzerland; 4 Danish Research Centre for Magnetic Resonance, Copenhagen University Hospital Hvidovre, Hvidovre, Denmark; 5 Department of Radiology, University of Wisconsin, Madison, Wisconsin, USA; 6 Copenhagen HIV Programme, Faculty of Health Science, University of Copenhagen, Copenhagen, Denmark

## Abstract

**Background:**

Bacteremia and systemic complications both play important roles in brain pathophysiological alterations and the outcome of pneumococcal meningitis. Their individual contributions to the development of brain damage, however, still remain to be defined.

**Methods:**

Using an adult rat pneumococcal meningitis model, the impact of bacteremia accompanying meningitis on the development of hippocampal injury was studied. The study comprised of the three groups: I. Meningitis (n = 11), II. meningitis with attenuated bacteremia resulting from iv injection of serotype-specific pneumococcal antibodies (n = 14), and III. uninfected controls (n = 6).

**Results:**

Pneumococcal meningitis resulted in a significantly higher apoptosis score 0.22 (0.18-0.35) compared to uninfected controls (0.02 (0.00-0.02), Mann Whitney test, *P *= 0.0003). Also, meningitis with an attenuation of bacteremia by antibody treatment resulted in significantly reduced apoptosis (0.08 (0.02-0.20), *P *= 0.01) as compared to meningitis.

**Conclusions:**

Our results demonstrate that bacteremia accompanying meningitis plays an important role in the development of hippocampal injury in pneumococcal meningitis.

## Background

Bacteremia and systemic complications are frequently associated with pneumococcal meningitis and, in approximately half of all fatal cases, are judged to be the primary causes of death [1,2]. Experimental pneumococcal meningitis studies have shown that accompanying bacteremia not only influenced mortality [3], but also the meningeal inflammatory response [4], cerebral autoregulation [5], and both ventricle size and brain edema [6]. Apoptosis in the dentate gyrus of hippocampus is an important histopathological finding in patients dying from bacterial meningitis [7], and in experimental meningitis, hippocampal apoptosis has been associated with the development of learning deficits (for a review see [8]). Both the invading pathogen [9] and host immune reactions [10,11] contributed to hippocampal injury during bacterial meningitis. Whilst it has been observed that systemically introduced pneumococci induced apoptosis in a non-meningitis sepsis model [12], the role of accompanying bacteremia on hippocampal apoptosis still remains to be defined in bacterial meningitis. Consequently, we investigated the role of bacteremia in the development of hippocampal apoptosis during experimental pneumococcal meningitis.

## Methods

All experimental protocols were approved by the Danish Animal Inspectorate. Meningitis was produced by intracisternal inoculation of ~3 × 10^4 ^colony forming units (CFU) *Streptococcus pneumoniae*, serotype 3 into the cisterna magna of anaesthetized (midazolam (1.88 mg/kg, Dormicum^®^) and fentanyl/fluanisone (0.12 mg/kg, Hypnorm^®^)) adult male Wistar rats (300-320 g in weight). The study was performed as part of a previously published magnetic resonance imaging (MRI) study [6].

The study was comprised of 3 experimental groups: I) Meningitis (n = 12). II) Meningitis with an attenuated bacteremia due to treatment with an iv injection of 4.5 g serotype-specific rabbit anti-pneumococcal capsular serotype 3 antiserum (Pneumosera^®^, Statens Serum Institut, Denmark) at time of bacterial inoculation (n = 14). III) Uninfected control rats (n = 8).

Cerebrospinal fluid (CSF) and blood samples were obtained 28 hours after bacterial inoculation and were analyzed for white blood cell (WBC) count using an automatic cell counter (Medonic CA620 VET, Boule Medical AB, Sweden) and for bacterial concentrations by plating 10-fold serial dilutions. A "disease severity score" included activity (0-4) and characteristics of eyes (0-2) and fur (0-2) as previously described in detail (i.e. 0 = normal; 8 = highest disease severity [13]). Rats were then sacrificed by an overdose of pentobarbital (Mebumal^®^, Nykomed, Denmark) at 28 hours after inoculation. However, 8 out of 14 rats having an attenuated bacteremia from therapy with serotype-specific antibodies were sacrificed at 38 hours due to a significant better clinical performance at 28 hours compared to the meningitis group (see below). All animals were perfused transcardially with 1.5% paraformaldehyde and their brains removed and stored in 1.5% paraformaldehyde prior to histopathological examination.

**Figure 1:**
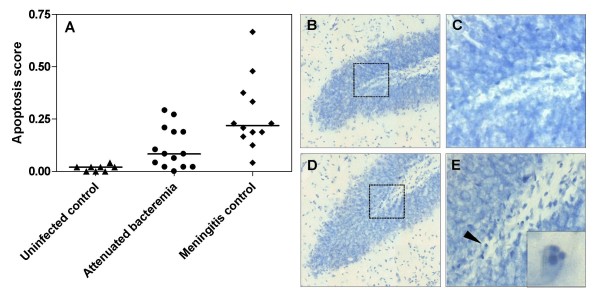
**Impact of bacteremia on hippocampal apoptosis in experimental pneumococcal meningitis**. A. Meningitis controls had significantly lower apoptosis scores* than uninfected controls and than meningitis rats with attenuated bacteremia due to treatment with serotype-specific antibodies (Mann Whitney, *P *= 0.0003 and *P *= 0.01, respectively). Bars represent medians. Hippocampal dentate gyrus histology showed sporadic occurrence of apoptotic cells (arrowhead) in rats with attenuated bacteremia (B+C) as compared to meningitis controls having a higher number of apoptotic cells (D+E). * Appearance of apoptotic cells were counted in 4 different slices spanning the hippocampus (3 visual fields in each of the two blades of the dentate gyrus). Each visual field was judged according to the following score: 0-5 cells = 0; 6-20 cells = 1; > 20 cells = 2. A mean value per animal was calculated from all inspected fields (48 fields per animal).

For the assessment of hippocampal brain damage, fixed brains were examined for the occurrence of apotosis in the dentate gyrus of the hippocampus. Cryosections (45 μm thick) were stained for Nissl substance with cresyl violet. Quantification of apoptotic nuclei in the hippocampal dentate gyrus was performed as described earlier [10]. In brief, cells exhibiting characteristic histomorphological features of apoptosis were counted in 4 different slices spanning the hippocampus of the right hemisphere. Three visual fields in each of the two blades of the dentate gyrus were inspected for the appearance of cells showing morphological signs indicative of apoptosis (condensed, fragmented dark nuclei, apoptotic bodies; Figure 1E). Each visual field was judged according to the following score: 0-5 cells = 0; 6-20 cells = 1; > 20 cells = 2. A mean value per animal was calculated from all inspected fields (48 fields per animal). Apoptosis was evaluated by a person blinded to the experimental grouping.

### Statistically analysis

All results are shown as medians with interquartile range. Comparisons between two groups were performed with Mann Whitney test. Correlation was performed with Spearman rank test. *P*-values less than 0.05 were considered significant.

## Results

As per the study design, blood bacterial concentrations at 28 hours after bacterial inoculation were significantly lower in meningitis rats treated with serotype-specific antibodies (0 Log_10 _CFU/mL (0-0.9)) as compared to untreated rats with meningitis (2.3 Log_10 _CFU/mL (1.8-2.9), Mann Whitney test, *P *= 0.001). Meningitis rats treated with serotype-specific antibodies also had a significantly lower disease severity score than untreated rats with meningitis at 28 hours (3 (2.8-3) vs. 4 (3-5), respectively, *P *< 0.05), whereas no significant differences in CSF bacterial concentration and in CSF and blood WBC were observed between the two groups. Uninfected controls had no signs of meningitis and a normal disease severity score (for details of laboratory data, see [13]).

As shown in Figure 1, untreated rats with meningitis had a significantly higher score of hippocampal apoptosis (0.22 (0.18-0.35) than uninfected controls (0.02 (0.00-0.02), Mann Whitney *P *= 0.0003), and when compared to meningitis rats with attenuated bacteremia due to treatment with serotype-specific antibodies (0.08 (0.02-0.20), *P *= 0.01). No significant difference in apoptosis scores was observed between meningitis rats with attenuated bacteremia that were euthanized at 28 hours or at 38 hours (0.13 (0.04-0.28) vs. 0.05 (0.02-0.15), respectively, *P *= 0.3), despite rats euthanized at 38 hours having a significantly higher disease severity score (5.0 (4.0-5.8)) than rats euthanized at 28 hours (*P *< 0.05).

The apoptosis scores correlated significantly with CSF bacterial concentrations, when all meningitis rats were analyzed together (n = 26, rho = 0.52, *P *= 0.008), but not when analyzed within each experimental group (untreated rats with meningitis: rho = 0.32, *P *= 0.33, and antibody treated rats: rho = 0.52, *P *= 0.06). In meningitis rats, no correlation was found between apoptosis scores and disease severity (rho = -0.09, *P *= 0.67), WBC in CSF (rho = 0.24, *P *= 0.24), and WBC in blood (rho = 0.10, *P *= 0.64).

## Discussion

In the present study we showed that accompanying bacteremia plays a significant role in development of apoptosis in the dentate gyrus of the hippocampus during pneumococcal meningitis. This is in line with our previous studies and emphasizes the importance of the systemic infection on pathophysiological alterations in meningitis such as the meningeal inflammatory response [4], cerebral autoregulation [5], and brain edema [6] as well as the outcome of pneumococcal meningitis [3]. Whilst several experimental meningitis studies have previously investigated the impact of various adjunctive therapies on the development of apoptosis (for a review see [8]), few reported blood bacterial concentrations [11,14]. Consequently, for those previous studies where blood bacterial concentrations were not reported, it remains largely unclear whether the anti-inflammatory treatment influenced the degree of bacteremia and subsequent development of hippocampal apoptosis.

Apoptosis in the dentate gyrus of hippocampus is an important histopathological finding in patients dying from bacterial meningitis [7], as well as in experimental meningitis, where hippocampal injury was correlated with the development of learning deficits (for a review see [8]). In contrary, no correlation between hippocampal volume loss (evaluated by MRI volumetric analysis [15]) and academic/behavioral limitation was demonstrated in surviving children with bacterial meningitis. Moreover, disturbance in memory has been correlated to the loss of cerebral volume and to the amount of white matter lesions in surviving adult meningitis patients using MRI [16]. However, this discrepancy could be explained by the relatively low resolution of the MRI studies suggesting that future high field and high resolution MRI studies still are warranted.

The exact mechanism by which bacteremia may influence the development of hippocampal apoptosis remains largely undefined. In accord with this study, Orihuela *et al. *showed that intravenous injection of pneumococci or pneumococcal cell wall caused neural apoptosis in a non-meningitis sepsis model [12]. Interestingly, the development of hippocampal injury was not secondary to systemic hypotension emerging during the bacteremia [12]. Also, cerebral ischemia from reduced cerebral blood flow/systemic hypotension occurring in experimental meningitis [5] did not significantly influence development of hippocampal injury, since therapy with antioxidants [17] and endothelin-receptor antagonists [18] failed to prevent hippocampal apoptosis, despite preservation of cerebral perfusion and prevention of cortical brain damage. In contrast, the systemic inflammatory response (e.g. sepsis, systemic inflammatory response syndrome) that may accompany bacteremia could play an important role. This is supported by mouse studies where IL-10 deficiency resulted in enhanced neural apoptosis, whilst IL-10 augmentation attenuated neural apoptosis following intravenous administration of pneumococcal cell wall [12]. However, we found no correlation between the hippocampal apoptosis score and the number of blood WBC, hence further studies are still required to investigate the influence of the systemic inflammatory response on hippocampal injury.

A limitation of the present study design was that not all 14 meningitis rats treated with serotype-specific antibodies were euthanized at 28 hours. Eight of these rats were euthanized at 38 hours in the aim to increase the disease severity score of rats with attenuated bacteremia and to mimic meningitis controls regarding disease severity. Since there was no significant difference in apoptosis scores between rats euthanized at 28 or 38 hours, this suggests that the results were not confounded by the time difference. Indeed, Gianinazzi *et al. *have previously shown, in a time course study over 168 hours, the time-point with maximal degree of hippocampal apoptosis in experimental pneumococcal meningitis (inoculum = ~10^5 ^CFU *S. pneumoniae*, serotype 3) was approximately 38 hours after infection [19].

## Conclusions

Our findings continue to underline the significant influence of systemic infection on both outcome and brain pathophysiology in pneumococcal meningitis including the development of hippocampal injury.

## List of abbreviations

CFU: colony forming units; MRI: magnetic resonance imaging; CSF: cerebrospinal fluid; WBC: white blood cell.

## Competing interests

The authors declare that they have no competing interests.

## Authors' contributions

CØ designed the study, analyzed the data, and drafted the manuscript. SL participated in the design of the study, performed histopathological analysis, and revised the manuscript. IR helped performing animal experiments and revised the manuscript. CB participated in the design of the study, performed the animal experiments, and revised the manuscript. All authors read and approved the final manuscript.

## Pre-publication history

The pre-publication history for this paper can be accessed here:

http://www.biomedcentral.com/1471-2334/10/1/prepub
